# Incorporation of NGR1 promotes bone regeneration of injectable HA/nHAp hydrogels by anti-inflammation regulation *via* a MAPK/ERK signaling pathway

**DOI:** 10.3389/fbioe.2022.992961

**Published:** 2022-09-23

**Authors:** Yi Liu, Yifan Zhang, Zexiang Zheng, Wenchao Zhong, Haiyang Wang, Zhen Lin, Lihua Li, Gang Wu

**Affiliations:** ^1^ Department of Oral Implantology, Guangzhou Key Laboratory of Basic and Applied Research of Oral Regenerative Medicine, Affiliated Stomatology Hospital of Guangzhou Medical University, Guangzhou, Guangdong, China; ^2^ Department of Material Science and Engineering, Engineering Research Center of Artificial Organs and Materials, Jinan University, Guangzhou, China; ^3^ Department of Human Genetics, Amsterdam UMC, Amsterdam, Netherlands; ^4^ Department of Human Movement Sciences, Faculty of Behavioural and Movement Sciences, Amsterdam Movement Sciences, Vrije Universiteit Amsterdam, Amsterdam, Netherlands; ^5^ Department of Orthopedics, Jinan University First Affiliated Hospital, Guangzhou, China; ^6^ Department of Oral Implantology and Prosthetic Dentistry, Academic Centre of Dentistry Amsterdam (ACTA), University of Amsterdam and Vrije Universiteit Amsterdam, Amsterdam, Netherlands

**Keywords:** bone regeneration, hyaluronic acid, nanosized hydroxyapatite, notoginsenoside R1, injectable

## Abstract

Suitable bone grafts are commonly required to achieve successful bone regeneration, wherein much effort has been spent to optimize their osteogenesis. Increasing evidence has demonstrated that reducing the levels of TNF-α can enhance bone regeneration at the injury site. Notoginsenoside R1 (NGR1) has been extensively studied in the field of anti-inflammation and regenerative medicine. Nanosized hydroxyapatite (nHAp) possesses excellent biocompatibility and osteoconductivity. In this study, we fabricated a thermoresponsive, injectable hyaluronic acid/nHAp (HA/nHAp) composite hydrogel incorporated with NGR1 to promote bone regeneration. Furthermore, NGR1-HA/nHAp hydrogel could enhance bone regeneration than those of HA and HA/nHAp hydrogels, profited by the underlying osteoblastic mechanism that NGR1 could facilitate activation of the MAPK/ERK signaling pathway and down-regulate the expression of TNF-α, ultimately upregulated expression of osteogenic genes. In summary, the NGR1-HA/nHAp composite hydrogel with controlled inflammation, and excellent osteogenic effect, will have great potential for use in bone regeneration applications.

## 1 Introduction

The repair of large bone defects caused by trauma, tumor resection, infection is still challenging in orthopedics (Wang et al., 2015a). Although autologous bone graft is considered as the gold standard for bone regeneration, it is often hampered by limited supply and painful at the donor site. Moreover, autologous bone often cannot appropriately match the size and shape of the defect areas (Wu et al., 2019). In recently years, tissue engineering and Various biomaterials scaffolds hold great promise for repairing bone defect. Hydrogels can form *in situ* to match the margins of bone defects and be shaped more easily than autologous bone, providing a foundation to guide bone regeneration (Kuang et al., 2019). Injectable, *in situ*-forming hydrogels are promising candidates for bone tissue engineering strategies (Ingavle et al., 2019; Zhao et al., 2019).

Hyaluronic acid (HA), one of the most used natural polymers for hydrogels, because of their structures similar to extracellular matrix of native tissues, possessing excellent biocompatibility and biodegradability (Garcia et al., 2019). However, they are often susceptible to mechanical disruption (Li et al., 2020). Thiolated hyaluronate has been utilized for forming hydrogels by reacting with electron withdrawing groups of other polymers through Michael addition. Here we present a thermo-responsive hydrogel with thiolated hyaluronate by introducing β-glycerophosphate disodium salt (β-GP) (Hansson and Lindman, 1996; Jin et al., 2010; Chen et al., 2018a). Since hydroxyapatite nano particles (nHAp) are biocompatible and osteoconductive, composites of HA and nHAp are expected to possess the favorable properties. Moreover, incorporation of nHAp into scaffold has also been shown to enhance mechanical strength and biomineralization (Dong et al., 2018; Fang et al., 2019; Zhou et al., 2019).

Bioactive factor is another important factor that accelerates the efficacy of bone regeneration (Lai et al., 2018). It has been reported that inflammation following implantation of bone grafts determines the success of bone regeneration (Sadowska et al., 2019). Tumor necrosis factor (TNF)-α is recognized as a crucial component of cytokines *in vivo* inflammation response. The high levels of TNF-α can impair bone regeneration (Sun et al., 2018). Recently, increasing evidences have demonstrated that reducing the levels of TNF-α can enhance bone regeneration at the injury site (Wang et al., 2015a). Notoginsenoside R1 (NGR1) is one of the main constituents of Panax notoginsen saponins (PNS), which has been extensively studied in the field of anti-inflammation and regenerative medicine. Our previous studies have demonstrated that NGR1 enhances osteoblastogenesis of MC3T3-E1 cells and accelerates matrix calcification (Liu et al., 2016). Moreover, NGR1 promotes osteogenic differentiation of osteoblasts by up-regulating the expression of ER signaling pathway (Wang et al., 2015b). NGR1 also has been widely applied in inflammatory diseases treatment. NGR1 protects PC-12 cells from LPS-induced damage and apoptosis by decreasing pro-inflammatory cytokines expression (Sun et al., 2019). Meantime, NGR1 alleviates TNF-α-stimulated Min6 and rat primary islets β cells apoptosis *via* activation of Wnt/β-catenin and PI3K/AKT/GSK3β signalling pathway (Chen et al., 2019). Therefore, incorporating NGR1 into HA/nHAp hydrogel to form injectable, *in situ*-forming composite scaffold is an attractive concept for developing biomaterials and might provide a method to enhance bone defect repair by reducing the inflammation reaction.

**SCHEME 1:**
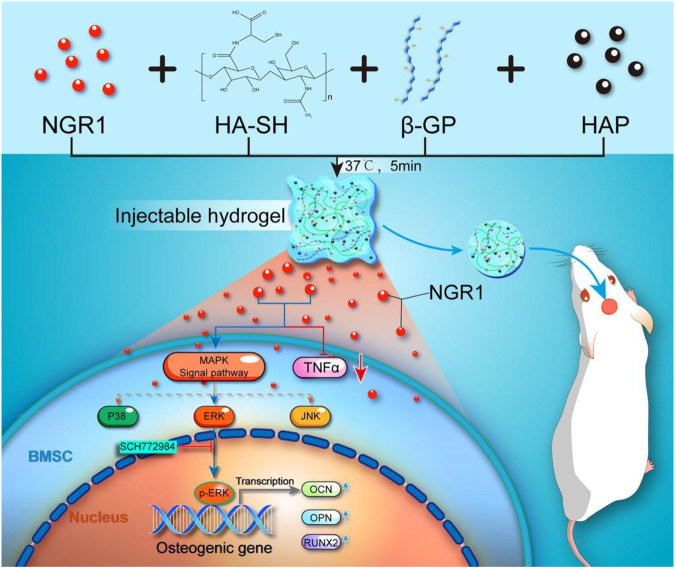
Schematic illustrations of preparation of a thermoresponsive, injectable hyaluronic acid/nanosized hydroxyapatite (HA/nHAp) composite hydrogel scaffold incorporated with Notoginsenoside R1 (NGR1) to promote bone regeneration. The underlying osteoblastic mechanism was investigated *in vitro* and revealed that NGR1 could upregulate the expression of MAPK/ERK signaling pathway and downregulate the expression of TNF-α, ultimately enhanced expression of osteogenic genes.

In the present study, as shown in Scheme 1, we fabricated an injectable NGR1-HA/nHAp composite hydrogel scaffold. A thiolated hyaluronic acid was firstly prepared, and was then cross-linked to form a gel by β-GP. The scaffolds with the selected suitable gelation parameters were characterized, and the mechanical strength was improved after co-blending with hydroxyapatite. The cytocompatibility of the scaffold was better and the addition of NGR1 showed osteogenic activity. The effects of this composite scaffolds on bone regeneration as well as inflammation were also studied using a rat model of cranial defects. In this study, hydroxyapatite and NGR1 synergistically created a microenvironment for bone tissue regeneration through hyaluronic acid microcarriers, and at the same time, NGR1 had a slowing effect on inflammation.

## 2 Materials and methods

### 2.1 Materials

Hyaluronic acid (HA, Huaxi Furuida Biomedical Co., Ltd.), L-cysteine hydrochloride monohydrate (L-cys, Sigma-Aldrich), 1-Ethyl-3-(3-Dimethylaminopropyl) Carbodiimide Hydrochloride (EDAC), N-Hydroxysuccinimide (NHS), and β-glycerophosphate disodium salt (β-GP) were purchased from Qi Yun Biotechnology Co., Ltd. (China). Nano hydroxyapatite (nHAP) was prepared according to the references (Li et al., 2011). NGR1 was purchased from Nanjing Zelang Medical Technological Co., Ltd. (ZL140310529, Nanjing, China). α-MEM and FBS were purchased from Gibco Inc. (Invitrogen, United States). All other reagents were of analytical grade and commercially available.

### 2.2 Synthesis and characterization of thiolated hyaluronic acid (HA-SH)

The method to synthesize HA-SH was modified according to our earlier publications (Li et al., 2017). In brief, 1.0 g HA was dissolved in 250 ml deionized water, EDAC and NHS was then added at a final concentration of 50 mmol/L. 1 mol/L HCl was dropwise added to adjust the pH of the reaction solution to 5∼6 and stirred for 30 min. Afterwards, 2.0 g L-cysteine hydrochloride monohydrate (L-cys) was added to the reaction system and 1 mol/L NaOH was added dropwise into the mixture until the pH was adjusted to 5.0. After reacting for 5 h under stirring, the product was dialyzed (MWCO 14 kDa) against HCl solution (pH = 5, containing 1 wt% NaCl), and finally freeze-dried. ^1^H NMR spectra of HA and HA-SH were obtained on an AVANCE III HD 600 NMR spectrometer (Bruker, Germany). The composition of the HA-SH and composite hydrogel with different precursor concentration was characterized by FT-IR with an attenuated total reflectance (ATR) accessory. The free sulfhydryl content of HA-SH prepared by Ellman’s reagent was determined by reference method (Li et al., 2017).

### 2.3 Preparation and characterization of the composite NGR1-HA/nHAp hydrogels

The weighed HA-SH was dissolved in deionized water to obtain a concentration of 4% (w/v). β-GP 8% (w/v) was then added with stirring to achieve a homogenous solution with the pH of about 7. Afterwards, nHAP and NGR1 (25 μg/μL) were dispersed in the solution with magnetic stirring of 500 rpm. The volume ratio of NGR1 solution to HA/nHAP solution was 1:3 (v/v) and the final concentration of nHAP in the dry nHAP/HA-SH composite was 10, 20, 30 and 50% (w/w), respectively. The hydrogel precursors were placed in a 37°C environment for gelation.

The gelation time was measured by vial inverting method and rheology test with a Kinexus Pro rheometer (Malvern, United Kingdom) equipped with a cone plate (φ 20 mm) at a frequency of 1 Hz and a strain of 1% in 37°C. The gels were subjected to a single compression and a cyclic multiple compression test by Universal material testing machine (American BOSE). The single compression test condition was 60% maximum deformation and 0.05 Hz frequency. Cyclic compression test performs 20 cycles of compression under the same conditions.

### 2.4 Cell proliferation and morphology assessment

The hydrogels were placed in a 96-well plate (1 sample per well) under sterile conditions. Cell proliferation on the hydrogel was evaluated by Cell Counting Kit-8 (CCK-8) assay. Briefly, cells were seeded in a 96-well plate at a density of 5,000 cells per well and incubated at 37°C for 1, 3 days. Then 10 μL CCK-8 solution and 90 μL culture medium were added to each well. The optical absorbance at 450 nm was detected using a spectrophotometric microplate reader (Thermo Fisher Scientific, United States). Then, cellular morphology was evaluated by Live-Dead kit. After culture for 1, 3 days, cells were washed by PBS for 3 times and incubated in 100 μL of Live-Dead solution for 30 min. Cellular morphology were observed under fluorescent microscope.

### 2.5 Osteoblastic differentiation and gene expression

#### 2.5.1 *In vitro* osteoblastic differentiation

The osteoblastic differentiation of BMSCs was evaluated in dissolution product of NGR1-HA/nHAp composite hydrogel. The extracted method has been used in previous studies (Lin et al., 2020). In brief, 100 mg freeze-dried hydrogels were immersed in 1 ml serum-free a-MEM medium to extract the NGR1. The liquid was collected and centrifuged after incubating for 24 h. Then the obtained NGR1 was purified by filtering the supernatant. 1 × 10^4^ BMSCs were seeded into a 48-wells plate and cultured for 3 days. Cells were then washed with PBS for three times and osteogenic induction medium containing BMP-2 (200 ng ml^−1^), with or without TNF-α (50 ng ml^−1^) were added. For anti-inflammatory therapy, cells were treated with extracted NGR1 in the presence of BMP-2 and TNF-α.

#### 2.5.2 ALP activity assay

Alkaline phosphatase (ALP) activity was measured using an Alkaline Phosphatase colorimetric assay kit (nanjing Jiancheng, China) according to the instruction. Briefly, 1.0 × 10^4^ cells/well were seeded and cultured with osteogenic induction medium in a 48-well plate for 7 days. Then, each sample was rinsed with PBS three times. The cells were lysed and ALP activity was measured by an ALP colorimetric assay kit (nanjing Jiancheng, China). The ALP activity was normalized by the total protein content that was measured using a BCA protein assay kit (Beyotime, China).

#### 2.5.3 ALP staining and alizarin red staining

Osteogenic differentiation was further evaluated by ALP staining and Alizarin red staining. BMSCs were cultured with osteogenic induction medium in 48-well plates. After 7 and 14 days of culture, cells were washed with PBS and fixed with 4% paraformaldehyde. Cells were then soaked in BCIP/NBT solution (Beyotime, Shanghai, China) and 1% Alizarin Red solution (pH = 4.1) for ALP staining and Alizarin red staining, respectively.

#### 2.5.4 OCN expression assay

OCN expression was used to further evaluate the osteogenic differentiation of the BMSCs. Cells were cultured with osteogenic induction medium for 14 days. Then the OCN expression was measured by a OCN EIA kit (Biomedical Technologies, United States) as previously described (Liu et al., 2016).

#### 2.5.5 Gene expression

The expression of the each gene was determined using a fluorescent qPCR Kit (Takara, China) in a real-time qPCR instrument (ABI 7500, United States). The mRNA expression of OCN, OPN and Runx2 were measured after 4 days of culture, and cells treated with nothing were employed as control group. All experiments were performed in three times.

### 2.6 Western blot analysis

Proteins were collected into RIPA buffer, and protein concentrations were detected by a BCA Protein Assay Kit (Beyotime, Shanghai, China). The proteins were separated by electrophoresis and transferred to PVDF membranes. After blocking in 5% nonfat milk, the membranes were probed with the primary antibody to P38, p-P38, ERK1/2, p-ERK1/2, JNK, p-JNK (Abcam, HK) or GAPDH (Abcam, HK) at 4°C overnight. Then, the membranes were washed three times and incubated with goat polyclonal secondary antibody to rabbit IgG-H&L (HRP) (Abcam, HK), followed by analysis using chemiluminescence imaging system. GAPDH was used as the internal control.

### 2.7 *In vivo* experiments

*In vivo* bone regeneration ability of NGR1-HA/nHAp composite hydrogel was evaluated by a rat cranial critical sized defect model (Sprague Dawley rats, male, 300–350 g). Briefly, the rats were anesthetized with 3% pentobarbital sodium solution. Critical sized defects were made using a 5 mm diameter trephine bur at the both sides of calvarium. The β-GP was then added to different composite hyaluronic acid solutions [HA (*n* = 6), HA/nHAp (*n* = 6) and NGR1-HA/nHAp (*n* = 6)] to prepare the resulting gel precursor solution, which was injected into the defect site *via* a syringe. Defects that were not filled were used as controls (*n* = 6). After 8 weeks, the rats were sacrificed. Then the samples were harvested and fixed in 4% paraformalclehyde for microCT analysis and histological evaluation. The defects were scanned by microCT (microCT80, Scanco Medical, Bassersdorf, Switzerland) at a resolution of 10 μm (80 kV, 100 μA) and then subjected to offline reconstruction (grey value: 0–0.075). To quantitatively evaluate the regenerated bone tissue within the defects, the bone mineral density (BMD), bone volume/total volume (BV/TV), and number of trabecular (Tb.N) was calculated. For the histology analysis, the harvested samples were fixed in 4% paraformaldehyde, decalcified in neutral 10% EDTA solution and embedded in paraffin. The samples were sectioned, then stained with HE and Masson’s trichrome. Immunohistochemical staining was used to evaluate the expression of TNF-α and OCN. Briefly, the samples were labeled with primary antibody solution containing anti-rat TNF-α (1:100, Servicebio, China) or anti-rat OCN (1:100, Servicebio, China) overnight. Then, the sections were stained with secondary antibody at room temperature and the nuclei were stained with HE.

### 2.8 Statistical analysis

All values are expressed as mean ± standard deviation (SD). All results were evaluated by one-way ANOVA using Graph Pad Prism 5. *p* < 0.05 was considered to be statistically significant.

## 3 Results

### 3.1 Properties of the hydrogels

**FIGURE 1:**
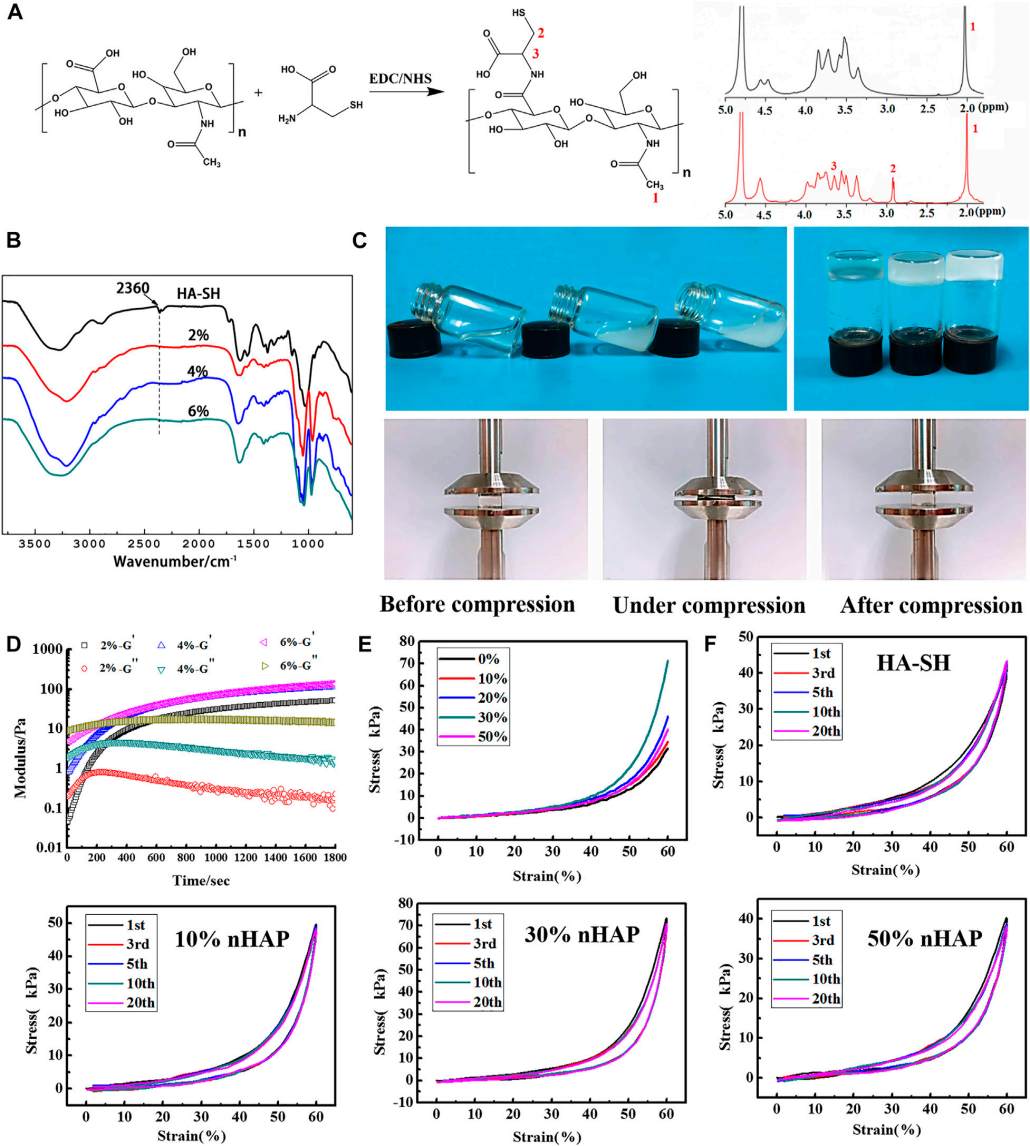
Characterization of the hydrogels. **(A)** 1H NMR spectra of HA and HA-SH; **(B)** FTIR spectra of HA-SH and composite hydrogel with different precursor concentration; **(C)** vial inversion to test sol-gel transition; **(D)** rheology study (37°C; frequency: 1.0 Hz; strain: 1.0%) **(E)** Compressive stress-strain curves; **(F)** cyclic compression test.

From Figure 1A, it was seen that the new peaks in ^1^H NMR spectra at δ = 2.91 ppm and δ = 3.65 ppm were due to the methylene protons of the -CH_2_CH_2_SH at HA-SH (He et al., 2013), and the free thiol groups was calculated as 316.67 ± 5.31 μmol/g. Figure 1B shows IR spectra of HA-SH and *in-situ* hydrogels were analyzed. The infra-red spectra of HA-SH and *in-situ* hydrogels. There is a S-H stretching vibration peak at 2,360 cm^−1^ for HA-SH. And the peak was absent after forming the gel, which proves that hydrogel is formed when the sulfhydryl group interacting on the side chain of HA. The new absorption peak at 968 cm^−1^ belongs to the characteristic absorption peak of phosphate group of β-GP. The fluid can be extruded through the needles except for the one with 50% nHAP due to its high viscosity. The gelation time was further tested by the inverting method (Figure 1C). As shown in Table 1, HA concentration had effects on the gelation time, and 4% was more suitable for the operation. There was no significant difference in the gelation time with the incorporation of nHAP and the gelation time remained 8–13 min for all the hydrogels (Table 1). The rheological test obtained by measuring changes in storage modulus (G′) and loss modulus (G″) with time. When the G′ intersected the G″, it indicated that the hydrogel network began to form. Most of the precursor showed the gelation starting points in the range of 100–200 s (Figure 1D). Three different concentrations of HA-SH hydrogels were prepared after freeze-drying. The microscopic morphology of the cross-sections was observed by SEM. The uniform pore structure of the hydrogel cross-sections (Supplementary Figure S1).

**TABLE 1:** The gelation time of hydrogels with the different HA and nHAP concentration.

HA concentration (wt (%)	HAP concentration (wt%)	Gelation time/min
2	0	20∼30
4	0	10∼12
6	0	3∼5
4	10%	8∼11
4	30%	11∼13
4	50%	9∼12

The rheological diagram of HA-SH + β-GP precursor was shown in Figure 1D, and β-GP and thiol groups were found to be two necessary components to form hydrogels in our study. In addition, we further studied five groups of precursors: HA-SH + β-GP, HA + β-GP, HA-SH, HA-SH + NaOH, and HA-SH + NaHCO_3_ at 37°C, and only the HA-SH + β-GP group formed a gel. The HA-SH rheological diagram (Supplementary Figure S2A) showed that the energy storage modulus and loss modulus do not intersect at the same time, and there is no gelation point. The rheological diagram (Supplementary Figure S2B) showed HA cannot form gel under the action of β-GP either. There is no gelation point. It is generally believed that the increase of pH value can promote the formation of disulfide bonds. We added NaHCO_3_ or NaOH in HA-SH aqueous solution with the same pH value as the β-GP, but it did not appear the gelation point (Supplementary Figures S2C,D), which means only weak alkalinity is not enough to break the hydrogen bond between HA-SH and water. The precursor of HA-SH, HA + β-GP, HA-SH + NaHCO_3_ and HA-SH + NaOH, was injected into the moulds, respectively, and placed in 37°C atm for 24 h. It was observed that these four groups did not transit to gel phase.

TEM images show that most of the nHAP are spherical shape and the particle size is between 100 and 200 nm (Supplementary Figure S3A). XRD reveals that the nHAP has a peak at 2θ = 31.6° (300) and a sharp characteristic peak at 2θ = 25.9 (002). Comparing with the HAP PDF card, the main crystal phase of the sample is HAP (Supplementary Figure S3B). The size of nHAP was also verified by the results of the Dynamic Light Scattering (DLS), which showed the average particle size was 164.2 nm, and the dispersion was uniform (Supplementary Figure S3C).

The single compression test results of the gels were shown in Figure 1E. The highest stress was 71.18 ± 1.38 kPa as the concentration of nHAP was 30%, double higher than that of the HA hydrogel. In addition, the hydrogel was resilient and could return to its original shape as removal of the force. Multiply cyclic compression test showed that the resilience of the composite hydrogel was improved with the addition of nHAP. After 20 cycles, the composite hydrogels were not damaged, and the stress-strain curves had a high coincidence (Figure 1F).

### 3.2 Cell viability

**FIGURE 2:**
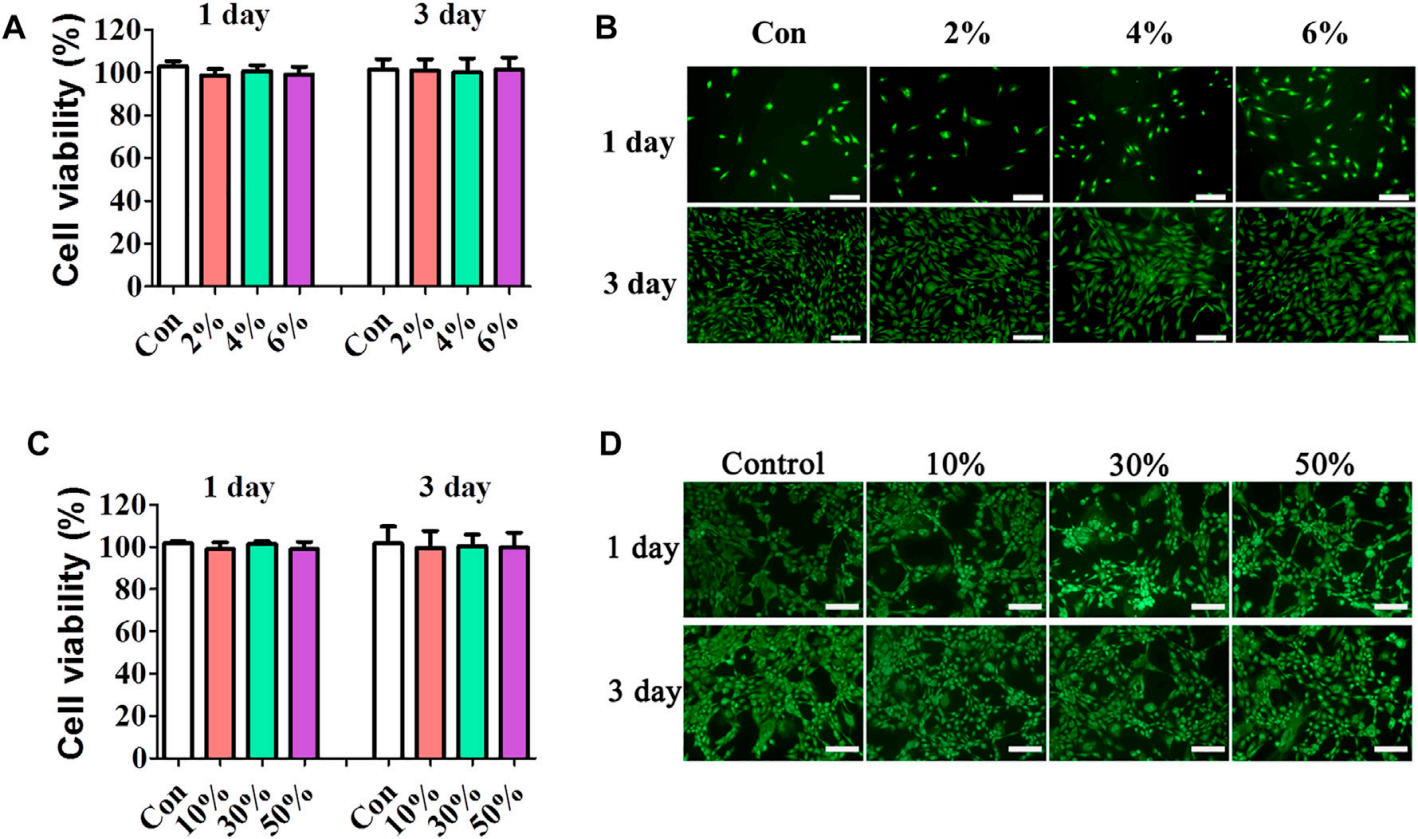
Cell viability and morphology on the composite hydrogels. Cell viability on different contents of HA hydrogels was evaluated by CCK-8 assay **(A)** and Live–Dead staining assay **(B)** (scale bar: 100 and 50 μm, respectively). Cell viability on different contents of nHAp hydrogels was evaluated by CCK8 assay**(C)**. **(D)** Cell morphology on different contents of nHAp was determined by Live–Dead staining assay (scale bar: 50 μm).

The cytotoxicity potential of hydrogels with different contents of HA was evaluated using CCK-8 assay. Figure 2A showed that all HA hydrogels did not show toxicity *in vitro* with all relative cell viability above 95%. Cell viability was further evaluated by live/dead staining. As shown in Figure 2B, very few dead cells were imaged in all HA hydrogels at day 1 and 3. Moreover, the cells were all well spread at day 3. These results indicated that composite hydrogels with different contents of HA had good cell biocompatibility.

The proliferation behavior of MC3T3E1 cells in composite hydrogels with different contents of nHAp (10–50%) was shown in Figure 2C. Results showed that the viability of cells on composite hydrogels showed no significant differences with that of the control group at 1 and 3 days (*p* > 0.05). As shown in Figure 2D, viable cells accounted for the majority in all composite hydrogels. Moreover, the viable cells on day 3 had remarkably higher density than that on day 1, demonstrating that cells have significantly proliferated in all composite hydrogels. These results showed that incorporation of nHAp has no adverse effects on the cell viability and growth.

### 3.3 Releasing behavior of NGR1 composite hydrogel

The release behavior of NGR1 from the composite hydrogel was evaluated, as shown in Supplementary Figure S4A. NGR1 had a burst release from the composite hydrogel in the first 12 h and plateaued within 72 h, with the release ratio of 62%. In addition, the residual of the NGR1 will be further released through the degradation of hydrogel. Therefore, the sustained release of NGR1 was observed from NGR1/nHAP/HA-SH composite hydrogel. As shown in Supplementary Figure S4B, the NGR1 released from the composite scaffold showed negligible cytotoxic effect on the cells.

### 3.4 The effect of NGR1 on osteoblastic differentiation of BMSCs treated with TNF-α

**FIGURE 3:**
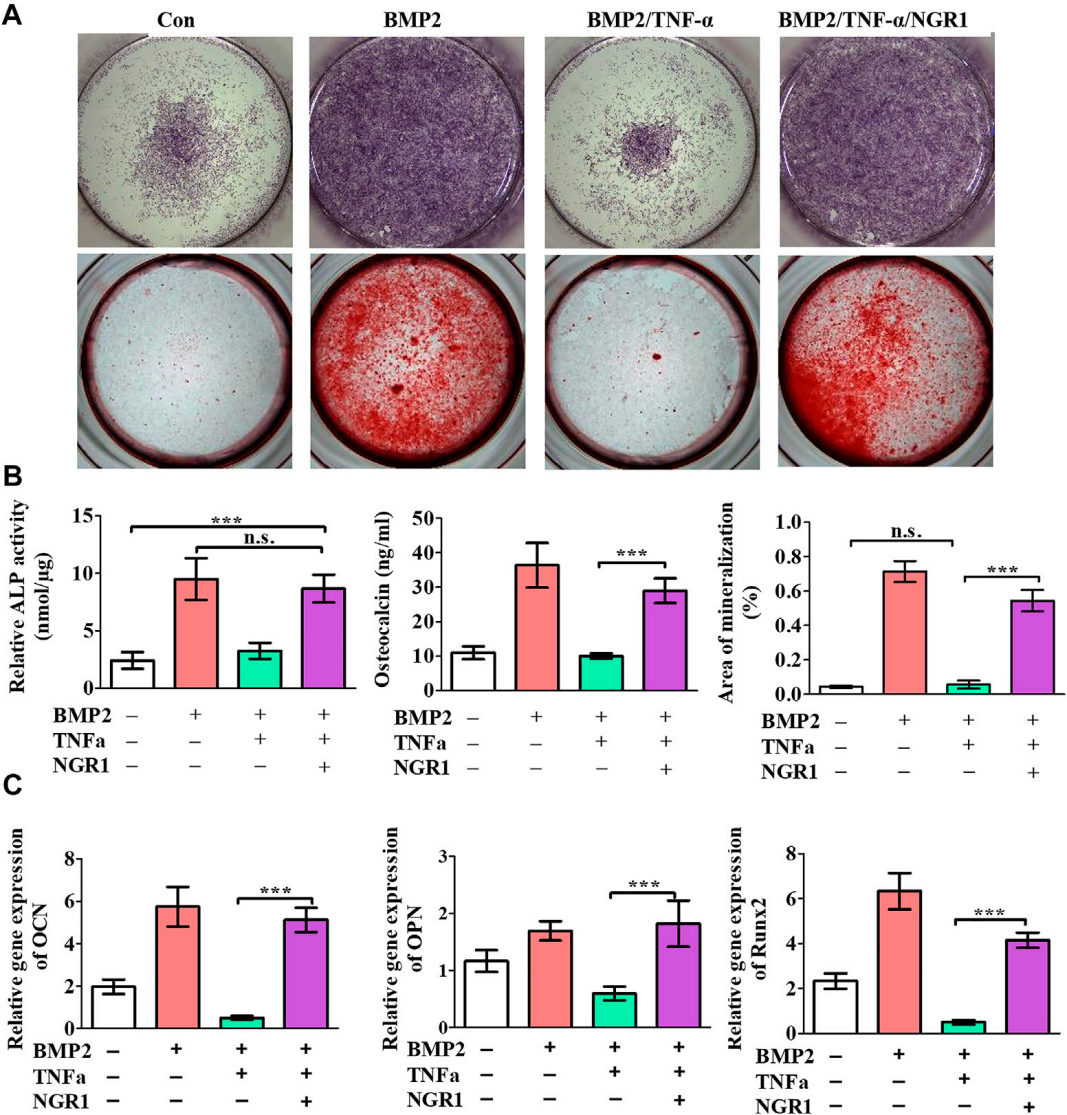
NGR1 promoted osteogenic differentiation of BMSCs treated with TNF-α and BMP2. **(A)** ALP staining and Alizarin red staining. **(B)** ALP activity, OCN expression and quantification of Alizarin red staining. **(C)** Relative mRNA expression of OCN, OPN and Runx2. (”*”: indicating *p* < 0.05, “**”: indicating *p* < 0.01, “***”: indicating *p* < 0.001).

TNF-α was shown to depress osteogenic differentiation capacity of stem cells. To investigate the effect of NGR1 on osteogenesis under TNF-α, the level of ALP and mineralized nodule deposition were analyzed. ALP staining showed that TNF-α attenuated BMP-2-induced ALP expression. However, the suppressive effect of TNF-α on ALP expression was reversed by NGR1 supplementation (Figure 3A). Alizarin red staining also demonstrated the protective effect of NGR1 on osteogenic differentiation in the inflammatory environment (Figure 3B). TNF-α reduced BMP-2-induced mineralized nodule deposition. However, these reductions were significantly attenuated in the NGR1-supplemented group (Figures 3A,B). To further determine these findings, the expressions of osteoblastic markers were analyzed by RT-PCR (Figure 3C). TNF-α could reduce the expression of the osteogenic genes. However, the inhibitory effect of TNF-α on osteogenic gene was reversed by NGR1 supplementation. These results further indicated that NGR1 can reduce the suppressive effect of TNF-α on BMP-2-induced osteoblastic differentiation.

### 3.5 Effects of NGR1 on MAPK signaling pathway

**FIGURE 4:**
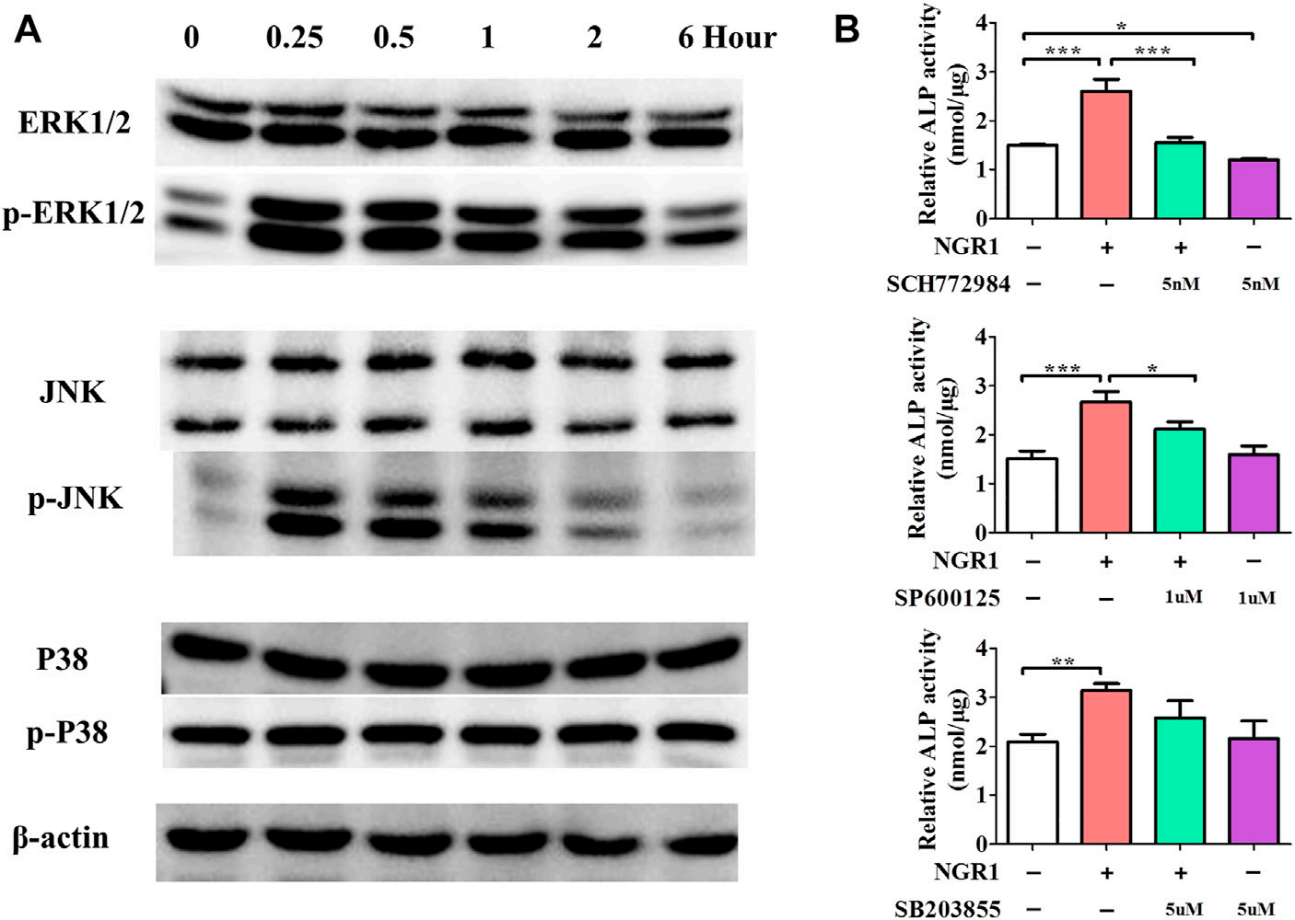
Effects of NGR1 on MAPK Signaling Pathway. **(A)** Western blotting analysis of protein expression of ERK1/2, p-ERK1/2, JNK, p-JNK, P38 and p-P38. **(B)** Effect of ERK, JNK and p38 inhibitor (SCH772984, SP600125, SB203580) on NGR1-induced ALP activity in BMSCs (”*”: indicating *p* < 0.05, “**”: indicating *p* < 0.01, “***”: indicating *p* < 0.001).

MAPK has been widely recognized as crucial signaling pathway in inflammation diseases and can promote osteogenic genes expression, consequently enhancing osteogenic differentiation of BMSCs. In order to illuminate the intracellular osteogenic mechanism, a MAPK signaling pathway was analyzed by Western blotting after the treatment of NGR1 (Figure 4A). The result demonstrated a significantly increase of p-ERK and p-JNK expression after 0.25 h. However, the expression of p-P38 was not changed. With blocking the ERK signaling pathway, ERK inhibitor (SCH772984) significantly blocked the effects of NGR1 on ALP activity (Figure 4B). However, NGR1-induced activation of ALP activity was only slight decreased by treatment of JNK inhibitor (SP600125). Results showed that blocking the ERK signaling pathway could suppress the ALP activity of BMSCs on NGR1. Therefore, ERK signaling pathway is necessary for NGR1 induced osteogenic differentiation of BMSCs.

### 3.6 Micro-CT measurement

**FIGURE 5:**
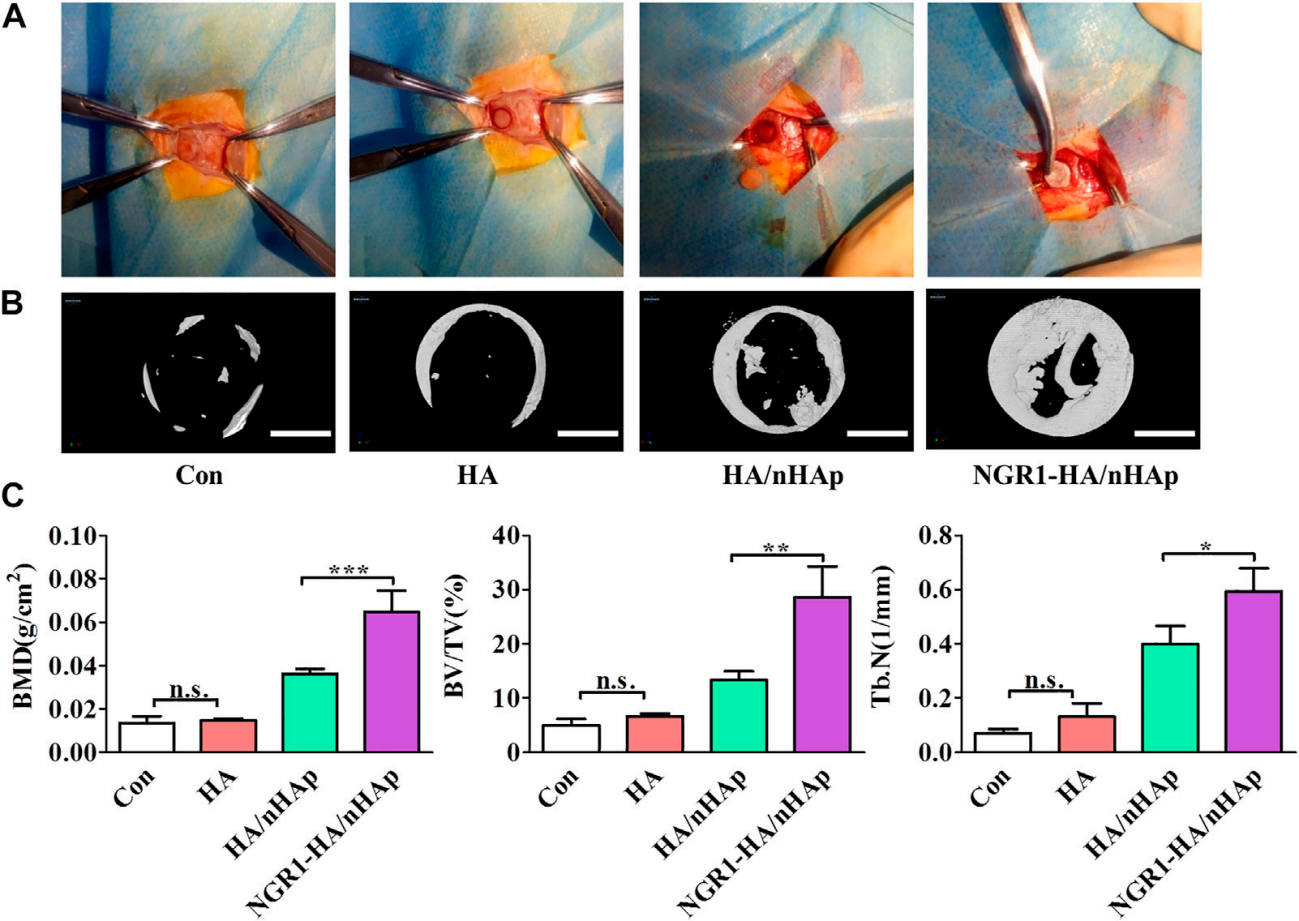
*In vivo* bone regeneration ability of NGR1-HA/nHAp hydrogel. **(A)** The procedure to establish a calvarial bone defect model and implant the composite hydrogels into the bone defect area. **(B)** MicroCT images showing the repaired calvarial defect 8 weeks post-implantation (Scale bars = 2 mm). **(C)** Quantitatively analysis of the BMD, BV/TV and Tb.N determined by micro-CT images. (”*”: indicating *p* < 0.05, “**”: indicating *p* < 0.01, “***”: indicating *p* < 0.001).

To evaluate the bone regeneration ability of the NGR1-HA/nHAp hydrogel *in vivo*, a calvarial bone defect model was established (Figure 5A), and then was analyzed by micro-CT analysis (Figures 5B,C). Micro-CT images showed that only a small amount of newly formed bone tissue could be found in Con, HA and HA/nHAp group in the bone defect area. However, large amount of new bone formation could be observed in NGR1-HA/nHAp group at 8 weeks after implantation (Figure 5B). More importantly, the quantitatively analysis demonstrated that NGR1-HA/nHAp group showed a significantly higher BMD, BV/TV ratio and Tb.N as compared with Con, HA and HA/nHAp group (Figure 5C) (*p* < 0.05).

### 3.7 Histological analysis

**FIGURE 6:**
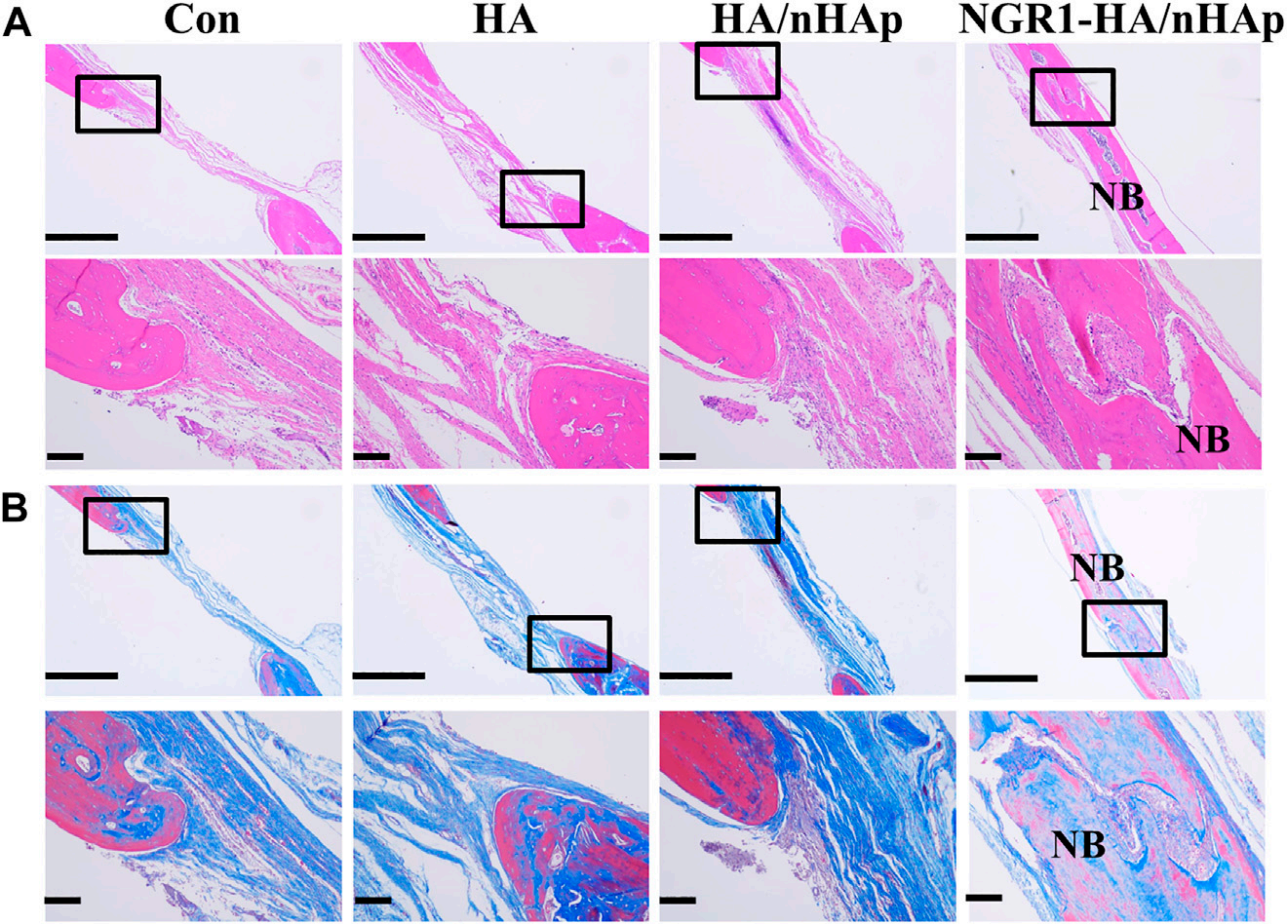
Histological analysis of new bone formation after 8 weeks of implantation *in vivo*. **(A)** H&E images. **(B)** Masson images (scale bar: 1,000 and 150 μm, respectively).

At 8 weeks, the harvested regenerated tissue at the defect were sectioned and inspected by histology analysis. Both H&E staining (Figure 6A) and Masson’s trichrome staining (Figure 6B) revealed that Con, HA and HA/nHAp group had little newly formed bone. However, much more newly formed bone was found in the NGR1-HA/nHAp group. In addition, there were negligible bone tissue in the Con and HA group, compared to a large amount of newly formed bone tissue in the HA/nHAp and NGR1-HA/nHAp group. NGR1-HA/nHAp group induced the best bone formation after 8 weeks of implantation *in vivo*.

### 3.8 Histomorphometrical analysis

**FIGURE 7:**
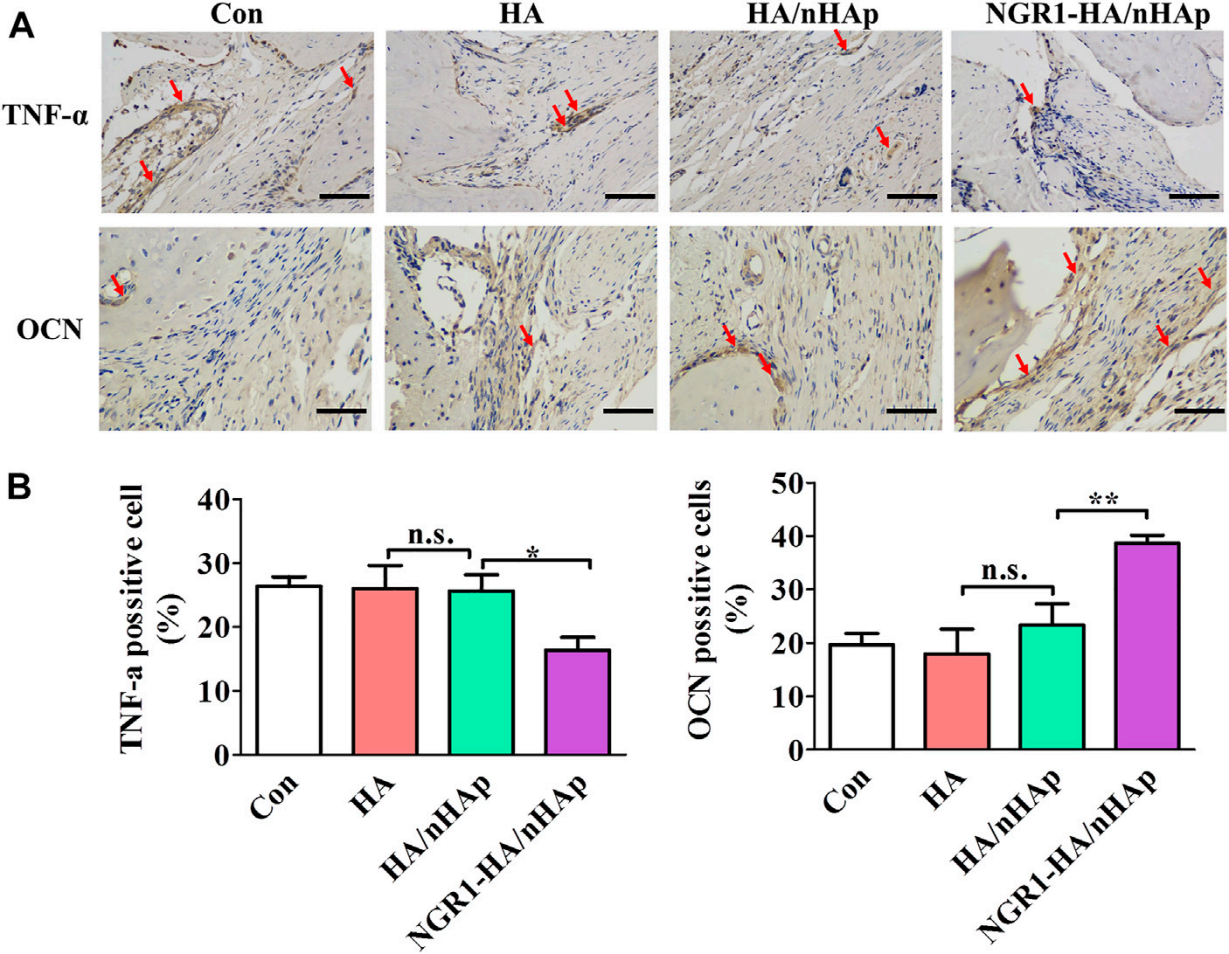
Immunohistological analysis of TNF-α and OCN for regenerated bone tissues. **(A)** Immunohistological images of TNF-α after 1 week of defect surgery and OCN after 8 weeks of defect surgery (scale bar: 100 μm). **(B)**. Number of TNF-α and OCN positive cells were calculated from the immunohistological images.

To investigate the effect of NGR1 on TNF-α expression, immunohistological analysis of TNF-α was also performed. As shown in Figures 7A,B, a large amount of TNF-α positive cells were detected in the Con, HA and HA/nHAp group. However, the number of TNF-α positive cell of the NGR1-HA/nHAp group was significantly lower than those of Con, HA and HA/nHAp group, demonstrating that NGR1 released from the composite scaffold inhibited the expression of TNF-α. To detect the effect of NGR1 on osteogenesis, OCN was further evaluated by immunohistochemical staining. As shown in Figures 7A,B, there were no statistically significant differences regarding the expression of OCN among the Con, HA and HA/nHAp group (*p* > 0.05). However, the OCN positive cells of the NGR1-HA/nHAp group were significantly higher than those of other groups (*p* < 0.05), thus indicating that NGR1 promotes osteogenesis.

## 4 Discussion

In this study, we fabricated thermoresponsive, injectable HA/nHAp composite hydrogels with controlled release of incorporated NGR1, capable of forming a gel *in situ* by body temperature triggering. The HA/nHAp composite hydrogel showed enhanced strength and suitable biocompatibility *in vitro*. In addition, NGR1 reduced the suppressive effects of TNF-α on BMP-2-induced osteoblastic mineralization. Furthermore, NGR1-incorporated hydrogel enhanced bone formation *in vivo* through activating MAPK/ERK signaling pathway.

Injectable hydrogels have aroused considerable interest in bone tissue engineering due to minimally invasive nature with which they can be delivered (Wu et al., 2019). The HA hydrogel was selected as scaffold in our study for two reasons. Firstly, it possesses suitable biocompability and biodegradation. Secondly, it can form *in situ* gelling system. Reports have already demonstrated the *in situ* hydrogel forming ability of HA (Zaviskova et al., 2018). HA-SH has been utilized to prepare hydrogels with other chemicals through chemical reactions between the functional groups. In this study, it was found that the HA-SH precursor was thermoresponsive and can form the gels *in situ*. β-GP and thiol groups were found to be two necessary components to form hydrogel. In addition, we studied five groups of precursors: HA-SH + β-GP, HA + β-GP, HA-SH, HA-SH + NaOH, and HA-SH + NaHCO_3_ at 37°C, and only the HA-SH+β-GP group formed a gel (Supplementary Figure S2). Studies have shown that as the temperature increases, the glycerol moiety of β-GP increases in hydrophilicity, which reduces the binding water of the hyaluronic acid molecular chain and promotes the interaction between the hyaluronic acid molecular chains. However, because this interaction is weak, it is not as stable as chemical bonds such as disulfide bonds. Therefore, pure hyaluronic acid does not form a gel under the action of β-GP. An alkaline environment promotes the formation of disulfide bonds. As the β-GP was changed to NaHCO_3_ or NaOH, but no gel formed. It may be because a large number of hydrophilic groups on the hyaluronic acid molecule bind to water molecules, and only weak alkalinity is not enough to break through hydrogen bonding, so that the sulfhydryl groups between the molecular chains react with each other. Therefore, β-GP promotes the formation of hydrogels in several ways. The glycerol moiety of β-GP, due to the large number of hydrophilic groups in its molecule, binds a large amount of bound water, resulting in a decrease in the binding water of the hyaluronic acid molecular chain, an increase in the interaction between the molecular chains, and an increase in the sulfhydryl group on the molecular chain. The mutual reaction also increases the hydrogen bonding of the intermolecular hydrophilic groups. The β-GP also has a -PO_4_^2−^ group, which can bind to H^+^ released by -SH, and promotes the formation of disulfide bonds. At the same time, the special structure of β-GP contains two hydroxyl groups in the molecule, and can also interact with hydrophilic groups on the molecular chain to form hydrogen bonds, and to link molecular chains, which also acts like a chemical crosslinker. In our study, 4% HA-SH hydrogel precursor showed the suitable injectability and viscosity.

However, the mechanical strength of the HA hydrogel is often insufficient for bone tissue engineering applications. Hydrogels loaded with inorganic particles, such as nano-hydroxyapatite (nHAp), have been reported to enhance mechanical properties and bioactivity (Wang et al., 2015a) (Ressler et al., 2018). In addition, incorporation of nHAp into hydrogel scaffold accelerated cell adhesion and osteogenic gene expression (Morsi et al., 2019). In our study, the synthesized nHAp had mean size of about 164.2 nm, and most of them were spherical particles (Supplementary Figure S3). The stress was significantly increased with different concentrations of nHAp incorporation, especially for the hydrogel with 30% of nHAP. However, the hydrogel with 50% nHAP showed excessive viscosity and its injectability was poor. It was demonstrated that the presence of nHAp in hydrogel had negligible cytotoxicity, promoted cell adhesion and proliferation *in vitro*. The biocompability was tested by seeding MC3T3-E1 cells on HA/nHAp surface. The results showed the HA/nHAp hydrogels support the cell adhesion and growth of MC3T3-E1 cells, confirming that these hydrogels possess excellent biocompability, and have no adverse effect on cell viability.

Bioactive factor is another critical factor for successful bone formation. Incorporation of bioactive factor into the composite scaffold greatly enhanced osteoblastic differentiation and mineralization potential of stem cells cultured on the scaffold. In fact, hydrogel, often used as a drug delivery system, has been designed to mediate a sustained release of bioactive factor. Moreover, bioactive factor was encapsulated in the hydrogel to protect them from enzymatic degradation *in vivo*. In our study, we fabricated an anti-inflammatory composite scaffold through incorporation of NGR1, which could induce a sustained release of NGR1 for 72 h. As shown in Supplementary Figure S4, the release rate was fast in the initial 12 h, and cumulative release got balanced after 72 h. The remaining NGR1 is still encapsulated in the hydrogel and acts as a sustained drug release. As the hydrogel degradation, the remaining NGR1 will be absorbed by cells.

TNF-α has become a hot topic in bone regeneration in the past decade. Previous reports demonstrated that high level of TNF-α in the bone defect impeded bone repair (Wang et al., 2015a). NGR1 has been demonstrated to attenuate the suppressive effects of TNF-α on BMP-2 induced osteogenesis *in vitro*. For the evaluation of the *in vivo* osteogenic potential of the bone repair materials, the NGR1-HA/nHAp hydrogel was implanted into bone defect of rat calvarial. Micro-CT images obtained from excised calvarial bones showed that the defect filled with NGR1-HA/nHAp hydrogel was almost fully repaired after 8 weeks. In comparison to HA and HA/nHAp hydrogel, NGR1-HA/nHAp hydrogel showed a markedly enhanced ability to regenerate bone tissue. As shown in (Figure 5C), the values of BMD, BV/TV ratio and Tb.N for NGR1-HA/nHAp hydrogel were significantly larger than that for the group treated with HA and HA/nHAp hydrogel. H/E and Masson trichrome staining images also confirmed that a larger amount of bone tissue formed at the defect treated with the NGR1-HA/nHAp hydrogel in comparison to those treated with HA and HA/nHAp hydrogel. All these results demonstrated that NGR1 released from the hydrogel can accelerate bone regeneration. Moreover, immunohistochemical images found that the numbers of TNF-α positive cell were significantly reduced in the NGR1-HA/nHAp group when compared with Con, HA and HA/nHAp group after implantation. In addition, significantly larger amount of OCN was detected in the NGR1-HA/nHAp group when compared with Con, HA and HA/nHAp group. These results demonstrated that NGR1 released from scaffolds may promote bone regeneration through down-regulating the expression of TNF-α.

The MAPK and its downstream signaling pathway ERK were further studied (Chen et al., 2018b; Wang et al., 2018). In this study, NGR1 could enhance the protein level of phosphorylated-ERK, showing a consistent trend with cell osteoinduction. With blocking the ERK signaling pathway, there was significant downregulation of ALP activity. These results showed that blocking the ERK signaling pathway could inhibit the osteogenic differentiation of NGR1, confirming the ERK signaling pathway took a dominant role in regulating the osteogenic differenatiation of NGR1.

## 5 Conclusion

Thermoresponsive, injectable hydrogels consisting of HA, nHAp and NGR1 were fabricated for bone repair. HA/nHAp composite hydrogels exhibited well-defined injectability and rapid gelation at 37°C. The NGR1-HA/nHAp hydrogel has negligible cytotoxicity. The released NGR1 can also reduce the suppressive effects of TNF-α on BMP-2-induced osteoblastic differentiation *in vitro*. Moreover, NGR1-HA/nHAp hydrogel facilitated activation of MAPK/ERK signaling pathway and suppressed the expression of TNF-α. Ultimately, the ostengenic genes (OPN, OCN, and Runx2) were up-regulated. Furthermore, *in vivo* results demonstrated that the NGR1-HA/nHAp hydrogel was able to fully repair critical-size calvarial bone defects in a rat model. In summary, the composite scaffold is an injectable and gel-forming scaffold at 37°C, with negligible cytotoxicity. On the one hand, NGR1 inhibits the expression of inflammatory factors, while positively contributing to bone tissue regeneration with nHAP. The composite NGR1-HA/nHAp hydrogel has great potential for translation to the clinic for bone repair.

## Data Availability

The original contributions presented in the study are included in the article/Supplementary Material, further inquiries can be directed to the corresponding authors.
