# Supplementary material for: Incorporation of NGR1 promotes bone regeneration of injectable HA/nHAp hydrogels by anti-inflammation regulation via a MAPK/ERK signaling pathway
Source: Front Bioeng Biotechnol. 2022 Sep 23;10:992961. doi: 10.3389/fbioe.2022.992961 (PMC9537692; doi:10.3389/fbioe.2022.992961)
Supplement: Supplementary file 2 [file DataSheet2.docx]

https://www.jianguoyun.com/p/Df12muAQ24DkChjbkc4EIAA
