# Supplementary material for: Incorporation of NGR1 promotes bone regeneration of injectable HA/nHAp hydrogels by anti-inflammation regulation via a MAPK/ERK signaling pathway
Source: Front Bioeng Biotechnol. 2022 Sep 23;10:992961. doi: 10.3389/fbioe.2022.992961 (PMC9537692; doi:10.3389/fbioe.2022.992961)
Supplement: Supplementary file 3 [file DataSheet1.docx]

**Supplementary Materials**

1. **Supplementary Methods**

**1.1 Hydrogel microscopic morphology observation**

The prepared hydrogel was placed in a 50mL centrifuge tube, frozen in a liquid nitrogen tank for 10min, freeze-dried and cut into small pieces using a scalpel. The samples were attached to a copper table with conductive adhesive, sprayed with gold and then observed by scanning electron microscopy for the shape of the hydrogel sections.

- 1. **Gelation of HA precursors**

Five groups of gel precursor were prepared: HA-SH + β-GP, HA-SH, HA+β-GP, HA-SH+ NaHCO_3_ and HA-SH+NaOH. The concentration of HA-SH or HA, and β-GP, was 4 % (w/v), 8% (w/v), respectively. The hydrogel precursors were stirred to achieve a homogenous solution with the pH of about 7, and then measured with a Kinexus Pro rheometer at a frequency of 1 Hz and a strain of 1% in 37 °C.

### In vitro release profile of NGR1

The release profile of NGR1 was measured as described previously[1]. Briefly, the NGR1/nHAP/HA-SH composite hydrogel was suspended in 3 mL release medium, and kept at 37 °C under constant agitation at 150 rpm. At each time point, 3 mL release medium collected and changed with the same volume of fresh medium. The collected media were analyzed by high performance liquid chromatography at a wavelength of 203 nm as described previously[2].

**2. Supplementary Figures and Captions**

**
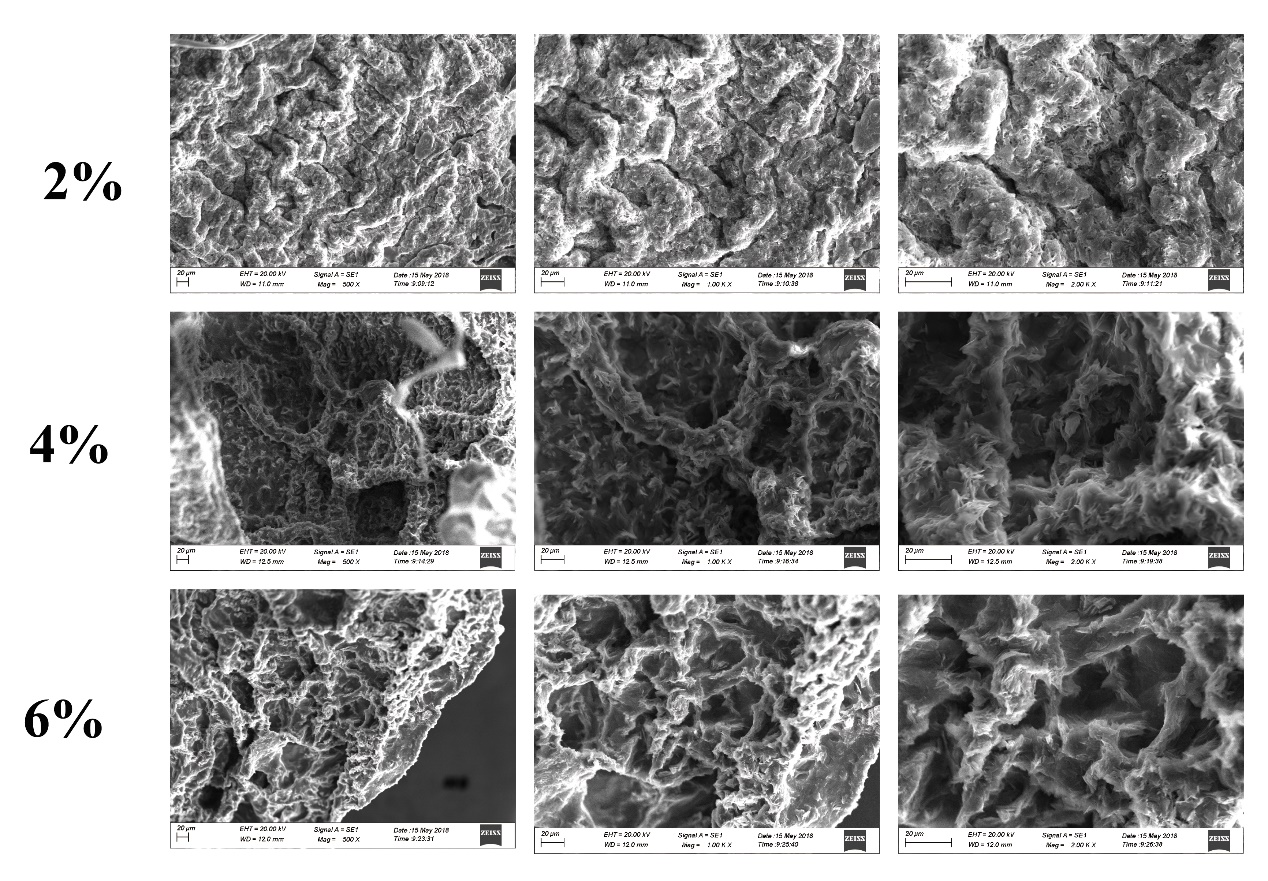
**

**Fig. S1** SEM images of different concentrations of HA-SH hydrogels


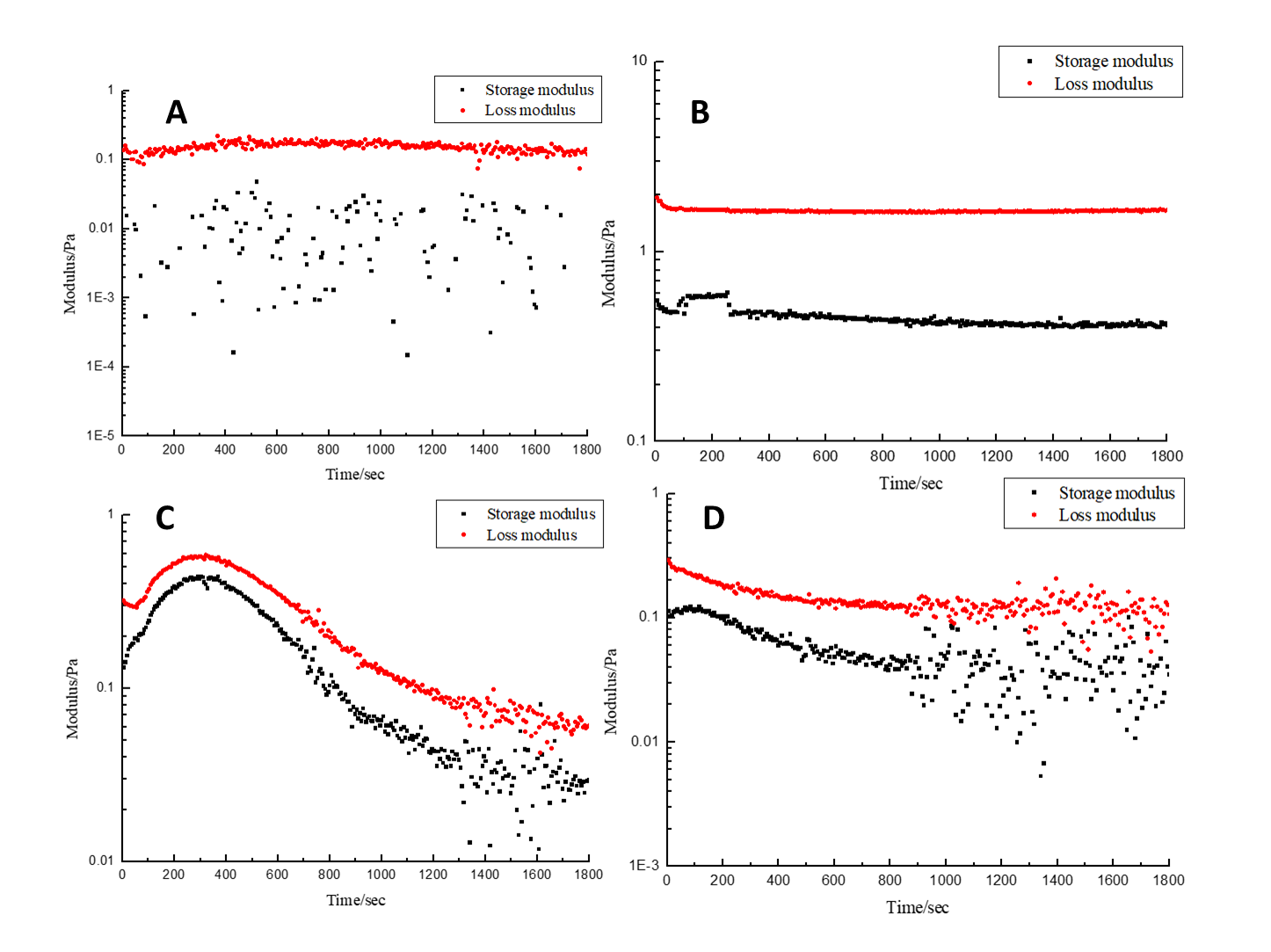


**Fig. S2** Rheological diagram of HA-SH solution (A), HA+ β-GP solution (B), HA-SH + NaHCO_3_ solution (C), and HA-SH + NaOH solution (D).


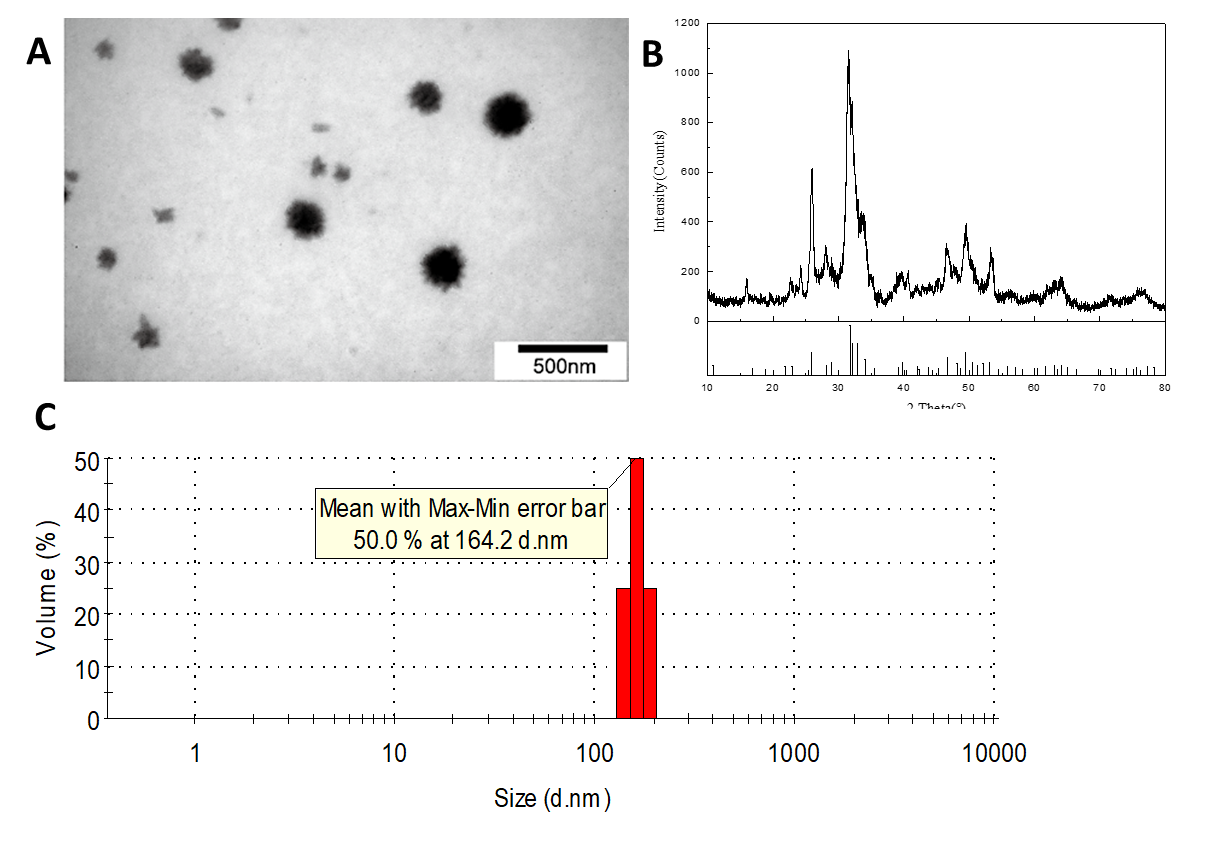


**Fig. S3** TEM image (A), XRD patterns (B), and particle size distributions of nHAP (C).


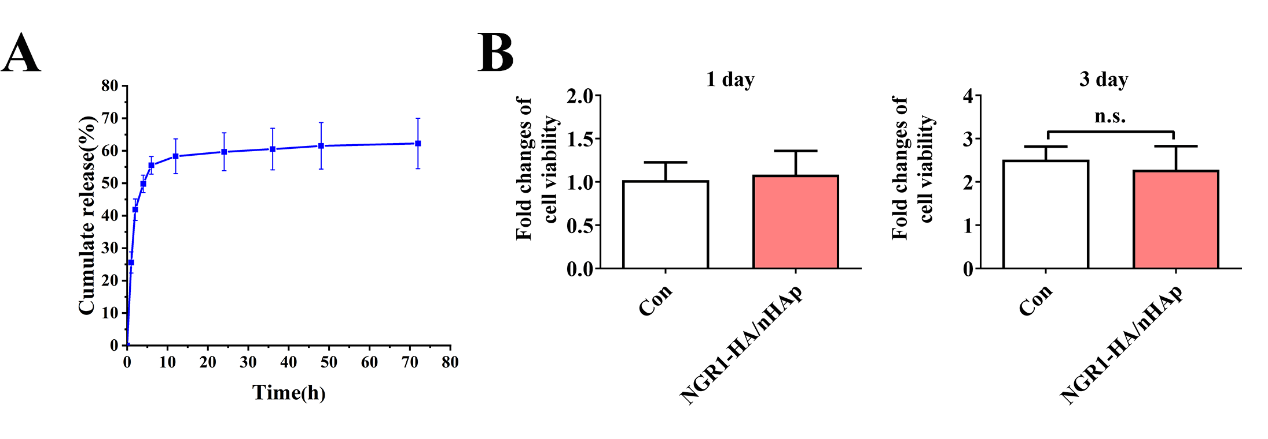


**Fig. S4** The sustained release of NGR1 from NGR1/nHAP/HA-SH composite hydrogel.

## References

[1] L.A. Zhen, C. Rlb, L.D. Yi, E. Yz, C. Na, W.A. Jing, B. Ll, W.F.J.C.P. Gang, Histatin1-modified thiolated chitosan hydrogels enhance wound healing by accelerating cell adhesion, migration and angiogenesis, 230.

[2] J.Q. Ruan, W.I. Leong, R. Yan, Y.T.J.J.A.F.C. Wang, Characterization of Metabolism and in Vitro Permeability Study of Notoginsenoside R1 from Radix Notoginseng, 58(9) (2010) 5770-5776.
